# Geometric constrains for detecting short actin filaments by cryogenic electron tomography

**DOI:** 10.1186/1757-5036-3-6

**Published:** 2010-03-05

**Authors:** Mikhail Kudryashev, Simone Lepper, Wolfgang Baumeister, Marek Cyrklaff, Friedrich Frischknecht

**Affiliations:** 1 Parasitology, Department of Infectious Diseases, University of Heidelberg Medical School, Im Neuenheimer Feld 324, D-69120 Heidelberg, Germany; 2 Center for Cellular Imaging and Nano Analytics (C-CINA), Biocenter, University of Basel, Mattenstrasse 26, CH-4058 Basel, Switzerland; 3 Department of Molecular Structural Biology, Max Planck Institute for Biochemistry, Am Klopferspitz 18, D-82152, Martinsried, Germany

## Abstract

Polymerization of actin into filaments can push membranes forming extensions like filopodia or lamellipodia, which are important during processes such as cell motility and phagocytosis. Similarly, small organelles or pathogens can be moved by actin polymerization. Such actin filaments can be arranged in different patterns and are usually hundreds of nanometers in length as revealed by various electron microscopy approaches. Much shorter actin filaments are involved in the motility of apicomplexan parasites. However, these short filaments have to date not been visualized in intact cells. Here, we investigated *Plasmodium *sporozoites, the motile forms of the malaria parasite that are transmitted by the mosquito, using cryogenic electron tomography. We detected filopodia-like extensions of the plasma membrane and observed filamentous structures in the supra-alveolar space underneath the plasma membrane. However, these filaments could not be unambiguously assigned as actin filaments. *In silico *simulations of EM data collection and tomographic reconstruction identify the limits in revealing the filaments due to their length, concentration and orientation.

**PACS Codes:** 87.64.Ee

## 1. Introduction

**Figure 1:**
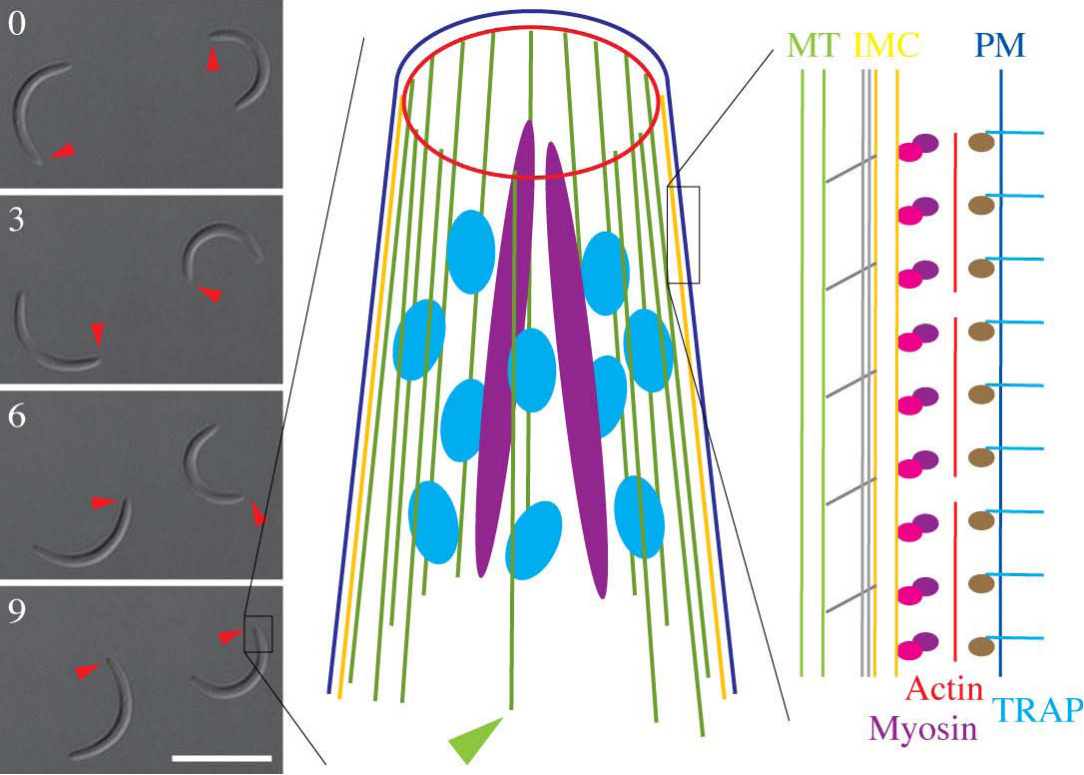
**Gliding motility of *Plasmodium berghei *sporozoites**. Four differential interference contrast images of sporozoites moving on a glass surface in a nearly perfect circular fashion and models illustrating the current understanding of sporozoite pellicle structure and molecules involved in motility. Scale bar: 10 μm. Arrowheads indicate the tip of the respective sporozoite and numbers indicate time in seconds. The black box indicates the apical (front) part of a sporozoite that is schematically illustrated in the cartoon. Shown are the polar ring (red) that organizes microtubules (green) and gates the secretion of rhoptries (magenta) and micronemes (light blue), the plasma membrane (dark blue) and the IMC (yellow). The green arrowhead points to the microtubule, which is localized away from the other microtubules. The black box at the right enlarges a view of the parasite pellicle to the right. The actin filaments (red) are located between the plasma membrane (PM, blue) and the inner membrane complex (IMC, yellow) and linked to trans-membrane receptors of the TRAP family (light blue) via the glycolytic enzyme aldolase (brown), while myosin (magenta) is linked via a complex of proteins (pink) to the inner membrane complex (IMC, yellow). The microtubules (MT, green) are linked to the IMC by linker molecules (grey). The long grey bars indicate the sub-pellicular network.

Actin filaments are important in many cellular processes including cell motility, cell division and organelle movement. Actin filaments (F-actin) are polymerized from monomers (G-actin) and their formation, disassembly and length is controlled by a large number of actin binding proteins [1]. Different arrays of actin filaments can be found in filopodia and lamellipodia, which are different structures important for cell motility [2]. Pathogens have found many ways of interfering with host cell actin dynamics to use this cytoskeletal component for their own end during invasion of or egress from a host cell [3,4]. In contrast to bacteria and viruses, parasites of the protist phylum of Apicomplexa, which are phylogenetically older than the common ancestor of animals and plants, can use their own actin cytoskeleton for motility through tissues and invasion of host cells [5,6]. Members of this phylum include malaria parasites (Plasmodia) and parasites causing Toxoplasmosis (Toxoplasma). In the invasive forms of these parasites the actin filaments are thought to be located in a narrow volume called the supra-alveolar space between the plasma membrane and an underlying alveolate-specific double membrane termed the alveoli or inner membrane complex (IMC) (Figure 1). The IMC is in turn connected to microtubules on its cytoplasmic face [7] (Figure 1). Actin is thought to be polymerized by a protein containing a formin homology domain [8]. Biochemical studies have shown that these actin filaments are very short, being only able to assemble *in vitro *into filaments of less than 150 nm in length [9-11]. Once polymerized these short filaments are thought to be linked via the glycolytic enzyme aldolase to a trans-membrane protein of the TRAP family, which spans the plasma membrane and thus links the parasite to its substrate [12-15]. An apicomplexa-specific myosin XIV has been shown to be anchored at the IMC and to provide the force for translocation [5,16,17]. Experiments with *Toxoplasma gondii *tachyzoites using different concentrations of drugs that interfere with actin dynamics revealed that the presence of actin filaments is rate limiting for motility in this form of the parasite [18]. Fractionation analysis showed that over 90% of actin is present in the monomeric form in both *T. gondii *and *P. falciparum *[18,19]. The trans-membrane proteins of the TRAP family are released by small vesicles (micronemes) at the front end of the parasite and cleaved by proteases in the plasma membrane near the rear end of the parasite [20,21]. This, together with the observation that small particles on parasite surfaces are translocated to the back end during gliding, supports the following model: actin filaments are nucleated at sites of contact between parasite and substrate at the front end. These filaments, anchored to the substrate via TRAP family proteins, are translocated backwards by myosin, thus pushing the parasite forward. Plasma membrane lipids and proteins are finally released into a membrane and protein rich trail that can be found behind parasites. In this manner parasites can move on a substrate for sustained periods of time [5,6,22] (Figure 1). It is important to note that different stages of different parasite species express different TRAP family members and show different types of motile behavior [5,23]. For example in the malaria parasite the red blood cell invading merozoites move only during host cell invasion [24], the mosquito gut invading ookinetes move slowly through several cells [25], while the sporozoites of the same species move much faster within the skin and liver [26]. One crucial cornerstone for all movement models is the dynamics of actin filaments. However, to date actin filaments have only been localized in intact parasites after treatment with actin filament stabilizing drugs [27].

Here, we aimed at visualizing actin filaments in *Plasmodium berghei *sporozoites using cryogenic electron tomography [28], which has readily revealed filaments in various cells types [29-32]. *Plasmodium berghei *is a rodent malaria parasite and sporozoites are the forms transmitted by the mosquito during a bite. Sporozoites move in a stick and slip fashion assisted by the formation and turnover of discrete adhesion sites, which regulate the overall speed of the parasites in an actin-dependent fashion [33]. We have used cryogenic electron tomography of sporozoites to report new features of the microtubule cytoskeleton and of the subpellicular network associated with the inner face of the IMC [7,34]. All these approaches imply that in principle the actin filaments should be detectable in the tomograms of sporozoites as well.

## 2. Results

**Figure 2:**
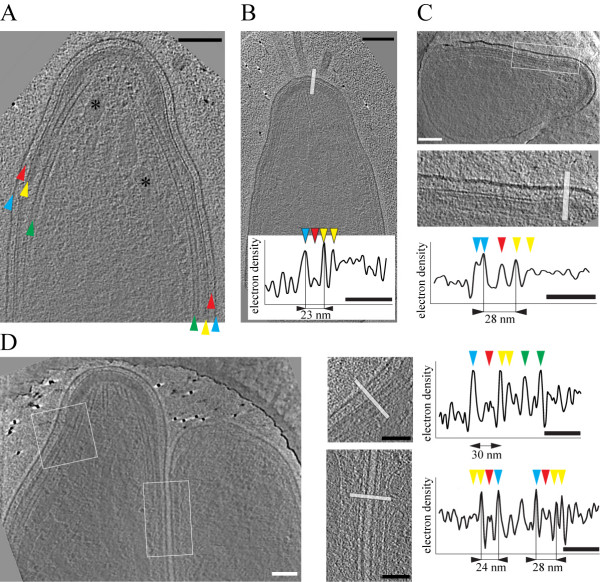
**Topology of the supra-alveolar space of *Plasmodium berghei *sporozoites**. A. A 10 nm thick slice through a tomographic reconstruction of the apical end of the sporozoite. Arrowheads indicate the plasma membrane (blue), the supra-alveolar space (red), the IMC (yellow) and microtubules (green). Two micronemes are indicated by asterisks. Note the electron dense cap that constitutes the polar ring (highlighted pink in Figure 3). Scale bar: 100 nm. B. A 10 nm thick slice through a tomogram of a different sporozoite. Scale bar: 100 nm. Graph depicts the electron density profile along the white bar. Arrowheads indicate the peaks corresponding to the plasma membrane (blue), material in the supra-alveolar space (red) and the membranes of the IMC (yellow). Scale bar: 50 nm. C. A 10 nm thick slice through the apical end of a different sporozoite showing a larger plasma membrane to IMC distance as the one in B. Scale bars: 100 nm for the micrograph, 50 nm for the electron density profile. D. A 10 nm thick section through a tomogram of a sporozoite that folds back on itself within a hole of the EM grid thus showing the apical and the rear end of the same parasite. White square and rectangle are enlarged to the right. The bars indicate the areas from which the electron density profiles were obtained. Peaks are indicated as in panel B with green arrowheads additionally pointing to the densities corresponding to microtubule walls. Note the different distances between the plasma membrane and the IMC as indicated. Scale bars: 100 nm for the micrographs, 50 nm for the electron density profiles.

*Plasmodium berghei *sporozoites move in circular patterns on flat supports (Figure 1) such as glass surfaces or coated EM grids [35-37]. Their form is preserved upon plunge freezing after which sporozoites can be imaged by cryo-electron tomography without the need for sectioning [7,34]. As actin filaments are thought to be located in the narrow supra-alveolar space between the plasma membrane and the IMC (the alveoli) and thus at the edge of the cell, one might expect to readily visualize these filaments. We investigated 50 tomograms taken from the apical end (11 tomograms), the proximal ends (9 tomograms) or the central region (30 tomograms) to analyze the electron density within the supra-alveolar space (Figure 2). This revealed a distance between the plasma membrane and the outer membrane of the IMC of 30 ± 6 nm (45 measurements from 40 different parasites) as measured between the centers of both membranes on electron density profiles across the parasite outer circumference (Figure 2). The minimal distance that we observed was 22 nm and the maximal distance was 44 nm. The average distance at the ends of the parasite was 29 nm, while in the central region it was 31 nm; however this difference was not significant (p = 0.11). The density profiles also revealed the presence of a discernable density in the supra-alveolar space, which could be found in all examined tomograms (Figure 2).

**Figure 3:**
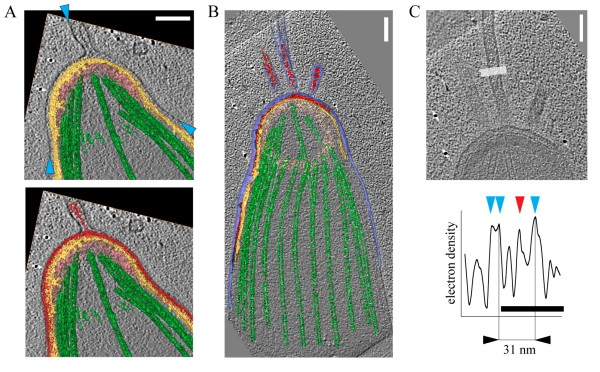
**Filopodia-like extensions of the plasma membrane at the apical end of sporozoites**. A. Volume rendered representation of the sporozoite shown in figure 2A overlaid with a slice through a tomogram showing an extension of the plasma membrane (blue arrowheads) with electron dense material inside (red). IMC (yellow), polar ring (pink) and microtubules (green) are highlighted. Scale bar: 100 nm. B. A volume rendered representation of the sporozoite from figure 2B showing four extensions of the plasma membrane with material inside. Scale bar: 100 nm. C. Enlarged view of the plasma membrane extensions from panel B and an electron density profile showing distinct peaks for the single leaflets of the plasma membrane (blue arrowheads) and the density inside the protrusion. Scale bars: 100 nm for the micrograph, 50 nm for the profile.

This density was particularly distinct on filopodia-like plasma membrane extensions observed in 3 of the 11 tomograms of the apical end (Figure 3). These extensions measured up to 500 nm in length and were between 31 and 39 nm wide. Similar to the supra-alveolar space, these thin projections could possibly accommodate up to 4 actin filaments if these were arranged in parallel and densely packed. However, despite the excellent signal to noise ratio as shown by the ability to resolve lipid bilayers in these thin projections, we found no evidence of any filamentous structures in the collectively two million nm^3 ^of volume that we analyzed in the tomographic reconstructions of these extensions.

**Figure 4:**
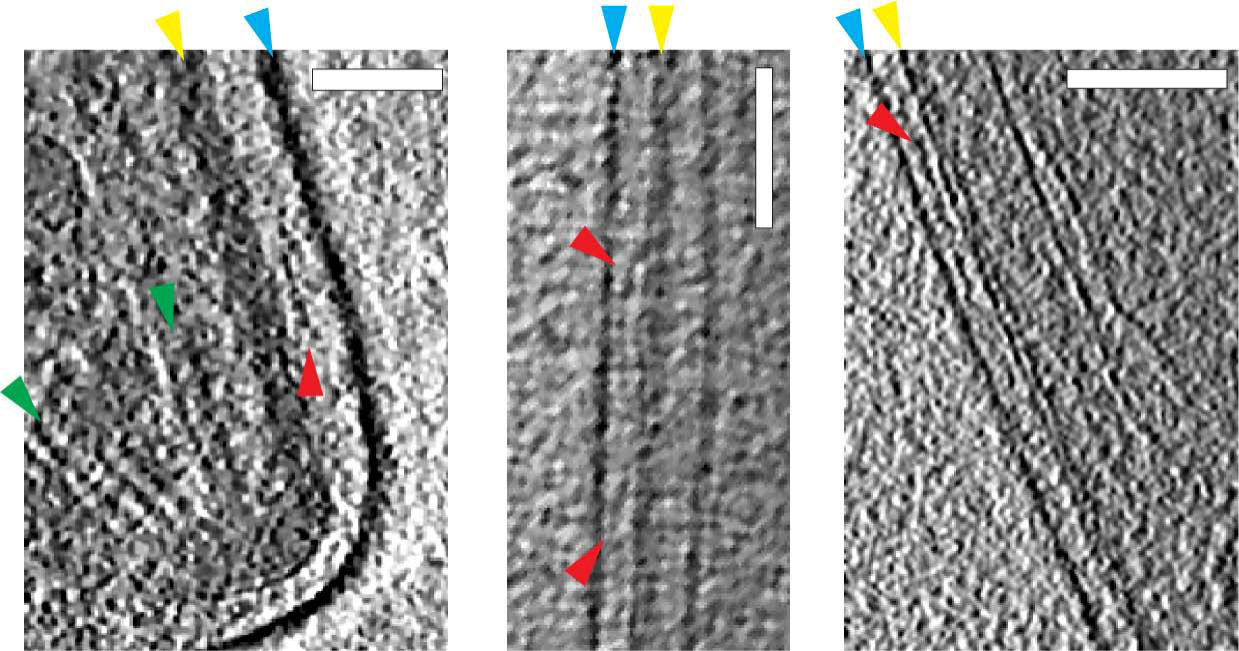
**Filament-like densities in the supra-alveolar space**. 10 nm thick sections through the tomograms from three different sporozoites showing the apical end (left panel), central region (middle panel) and the proximal end (right panel). Small filamentous structures (red arrowheads) between the plasma membrane (blue arrowheads) and the IMC (yellow arrowheads) are highlighted. Green arrowheads point to microtubules in the left panel. The direction of the electron beam is perpendicular to the plane of the sections. Scale bars: 100 nm.

Somewhat in contrast to the situation in the filopodia-like extensions, close investigations of the much larger volumes of the supra-alveolar space revealed a number of filament-like structures (Figure 4). These filamentous structures were between 20 and 200 nm long, either straight or undulating and could be found in the apical, central and proximal regions of the parasite (Figure 4).

**Figure 5:**
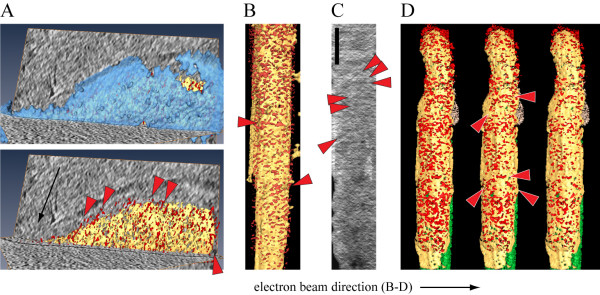
**Side views of the supra-alveolar densities reveal some filamentous structures**. A. Two views onto the plasma membrane (blue, top) and the IMC (yellow) from the same tomogram. The supra-alveolar densities are highlighted in red, and the red arrowheads indicate potential filaments. Note that most filaments are in parallel to the direction of the electron beam (black arrow). B. A volume rendered visualization of a side view onto the IMC. Red arrowheads point to the filament-like densities. The horizontal direction of the electron beam is indicated with a black arrow below panels B-D. C. A 10 nm thick section of a tomogram from a different sporozoite viewed from the side onto the IMC. Red arrowheads point to the filament-like densities. Scale bar: 100 nm (for B-E). D. Volume rendered representations of the density between the IMC and the plasma membrane for the tomogram from figure 2B with three different levels of threshold applied for visualization of the particular voxels (left panel - highest, right panel - lowest). Red arrowheads in the middle panel indicate filamentous structures. The direction of the electron beam is indicated with the black arrow below the panel.

Next, for better visualization we computationally segmented the volumes of the tomographic reconstructions corresponding to the IMC, the plasma membrane and the densities between these membranes in the supra-alveolar space (Figure 5). This revealed a number of delicate filamentous structures that were mainly oriented along the tomographic z-axis i.e. in the direction of electron beam (Figure 5B, C). These structures might be real or could be caused by the anisotropic resolution in the tomograms, which can artefactually alter the interpretation of fine structural features due to e.g. the smearing of densities in one direction, typically along the incident electron beam. The missing wedge effect is an intrinsic limitation of imaging in electron microscopy [28]. Such filamentous structures also appeared at different angles to the electron beam. All these filaments could be revealed at different threshold levels used for visualization (Figure 5D). Nevertheless, these densities and those shown in figure 4 could not be unambiguously assigned as actin filaments without further evidence. In contrast, we readily found actin filaments in cultivated mammalian cells such as Ptk2 cells and neurons [31,32] that were recorded under the essentially same imaging conditions. In addition we detected cytoskeletal filaments with dimensions similar to actin filaments in bacteria causing Lyme disease [38].

**Figure 6:**
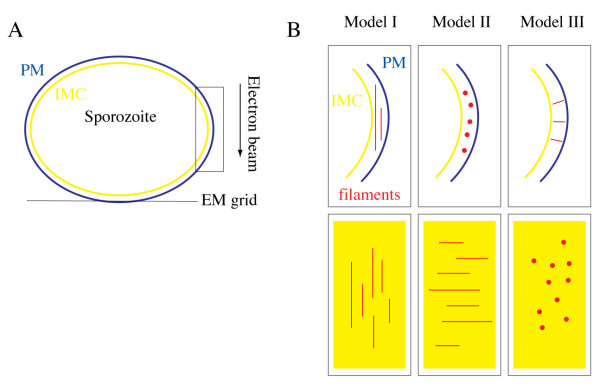
**Orientations of filaments used for simulation experiments**. A. The plasma membrane (blue) and IMC (yellow) of a slightly flattened sporozoite resting on an EM grid are shown. The arrow indicates the direction of the electron beam. The black box indicates the area enlarged as side and top views in B. B. Cartoons illustrating the orientation of model filaments used for simulation experiments in figures 7 and 8. The red lines and dots represent side and top views of filaments, respectively. The top row shows views along the parasite axis. The bottom row shows views from the plasma membrane onto the IMC. Model I: filaments perpendicular to sporozoite axis and parallel to electron beam direction. Model II: filaments parallel to sporozoite axis and perpendicular to electron beam direction. Model III: filaments perpendicular to sporozoite axis and perpendicular to electron beam direction.

**Figure 7:**
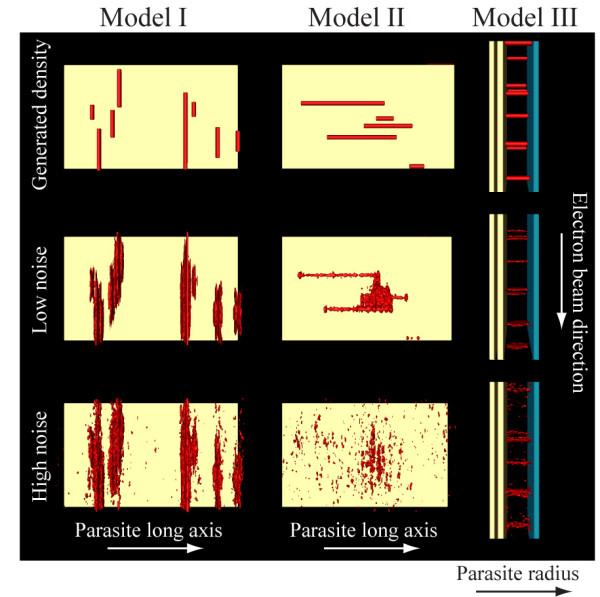
**Different orientations of *in silico *simulated filaments**. Left column: filaments with an average length of 48 nm are oriented in the direction of the electron beam and perpendicular to the parasite long axis corresponding to model I in figure 6. Middle column: filaments with an average length of 48 nm are oriented perpendicular to the electron beam and parallel to the parasite long axis, corresponding to model II in figure 6. Right column: filaments of 30 nm length connect the IMC and the plasma membrane (blue) and are viewed perpendicular to both the parasite long axis and the electron beam, corresponding to model III in figure 6. The noise in simulated tomograms increases from top to bottom. Note that filaments can be seen if oriented perpendicular to the parasite long axis, which is the tilt axis.

**Figure 8:**
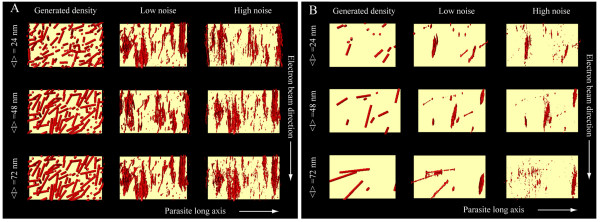
**Simulation of tomographic reconstruction for randomly oriented filaments**. Analysis of filaments (red) at high (A) and low (B) concentrations viewed over the IMC (yellow). Filaments of three different mean lengths were generated: 24, 48 and 72 nm (left column) and affected by low (middle column) and high (right column) noise; the tilting was performed around the parasite long axis (horizontal), the direction of the simulated electron beam was vertical. Note that longer filaments can be more reliably distinguished at lower noise (B, bottom row, central panel).

As actin filaments in *Plasmodium *and the related apicomplexan parasite *Toxoplasma gondii *were reported to be very short when polymerized *in vitro *[9-11] we next wondered whether there exists a size limit for the detection of actin filaments by cryogenic electron tomography. To this end we reasoned that simulation experiments similar to the ones we recently performed for the analysis of microtubules [34] could yield an answer. We therefore generated *in silico *populations of filaments with 24 to 72 nm in average length and a diameter of 5 nm (Figures 6 and 7, see also materials and methods). At this length the filaments could be placed in any direction parallel to the plane of the IMC into the supra-alveolar space. For simulations we concentrated on the volumes of the parasite periphery along the x and y tomographic axes (box in Figure 6A), rather than the z-axis, which is affected by the missing wedge and smearing of densities, which makes it difficult to accurately localize the pellicular membranes. For analysis we focused on the orientation of the filaments placed either perpendicularly or in parallel to the major tomographic axes (Figure 6B). The noise was added at levels comparable with those measured in electron tomograms of intact cells (Figure 7). This confirmed that filaments oriented perpendicularly to both the electron beam and the tilt axis smear stronger and thus disappear faster with increasing noise than these oriented otherwise. Indeed at high noise to signal (i.e. low signal to noise) ratios the densities smear so exceedingly in the direction of the electron beam that they may be detected as artefactual filaments (Figure 7, Model II, bottom panel). Filaments that connect the IMC with the plasma membrane are oriented perpendicular to both the tilt axis and the direction of the electron beam (Figure 7 Model III). Curiously, the simulations showed that such filaments, which are necessarily much shorter, could be detected reliably even at high noise levels. We next analyzed randomly oriented filaments with similar noise added to the models (Figure 8). Varying the concentration, the preferred length of actin filaments and the applied noise showed that those filaments that were located at larger distances from each other (i.e. at low concentrations) could be reliably visualized in the presence of moderate but not high noise (Figure 8). As expected, longer filaments could more readily be detected than shorter ones. Filaments oriented in the direction of the electron beam (vertically in Figure 8) appeared more pronounced. Taken together the simulation experiments suggest that the observed scarcity of filaments in cryogenic electron tomograms of sporozoites could be due to a combination of their short length, a too high concentration of filaments, the orientation of the filaments parallel to the parasite long axis, or the high noise. Alternatively, there could have indeed been no filaments present in the investigated volumes.

## 3. Discussion

Only few studies report the presence of actin filaments in apicomplexan parasites despite the clear indication that actin is important for parasite motility and host cell invasion [5,39]. In *Toxoplasma*, actin filaments were first reported from high-resolution low-voltage field emission scanning EM studies [40]. A subsequent study showed that actin filaments are rate limiting for *T. gondii *tachyzoites motility and the filaments were visualized as parallel arrangements in freeze-dried platinum replicas [18]. As expected filaments were not detected after application of cytochalasin D, an F-actin depolymerizing drug, while randomly arranged filaments could be found after application of jasplakinolide, an inhibitor of actin filament disassembly, which leads to disordered actin filament arrays [41,42]. As the core machinery that drives parasite motility and invasion is conserved between *Toxoplasma *and *Plasmodium*, it appears likely that filaments are arranged in a similar fashion in *T. gondii *tachyzoites and *Plasmodium *sporozoites [5]. We detected sparse filamentous structures of 20-200 nm length in the supra-alveolar space of *P. berghei *sporozoites. However, due to their undulating shape and rare appearance we cannot categorize them unambiguously as actin filaments. The question raises why cryogenic electron tomography, an imaging method that is exceptionally suitable for analyzing F-actin in whole intact eukaryotic cells [29-32] and has also revealed filaments in bacteria [38,43-46] failed to visualize actin in *Plasmodium *sporozoites.

In the following paragraphs we discuss four possible reasons for this failure. One possibility is that the regulation of filament formation and its turnover, and thus filament length varies between the two apicomplexan parasites. Although little is known about parasite or stage specific effects of actin-binding proteins [47] an interesting recent study showed that the beta-subunit of the actin capping protein is important for ookinete and essential for sporozoite motility, while being dispensable for merozoite invasion [48]. Also, a number of trans-membrane proteins of the TRAP family that play important roles in parasite motility are unique to the respective parasites and parasite forms [23,49,50].

A second possibility could be the reported shortness of actin filaments [9-11,18]. Indeed, simulation experiments showed that actin filaments could be short enough to fall below the detection limit of cryo-electron tomography. Simulation experiments showed that short filaments will be most reliably detected if oriented in the direction of the electron beam and perpendicularly to the tilt axis (Figure 7 and 8). In our tomograms this ideal situation would be given if filaments link the IMC with the plasma membrane (Figure 7). However we found no such connectors, suggesting that either the filaments are less than 30 nm long, or we underestimated the level of noise for our simulations, or that this somewhat unexpected orientation does indeed not occur in sporozoites. Simulation experiments also showed that filaments oriented in the expected way in parallel to the IMC and plasma membrane are harder to detect if they are not oriented in the direction of the electron beam. This was the case for the filament-like structures described in figure 4, which could have thus been tempting to accept as true actin filaments. Such filaments, oriented perpendicular to the beam can give rise to the appearance of wrongly (90° tilted) oriented filaments that only appear in the direction of the electron beam when high noise is applied for the models (Figure 7). Lastly, amongst a high concentration of densities of different shapes it would be very hard to detect single filaments (Figures 7 and 8). For these reasons we cannot unambiguously define the detected filamentous structures as filaments, whether made from actin or other proteins. In contrast, long filaments can be readily detected by eye and by surface rendering algorithms. Obviously, both types of filaments are equally affected by the noise in tomograms; however, long filaments are suggestively easier to detect and trace. This "eye catching effect" is similar to what had been extensively studied in the early days of electron microscopy. Then, studies comparing point-to-point, line-to-line or plane-to-plane resolution in EM images indicated that the latter yielded the highest accuracy. Clearly, for reliable detection one would ideally employ independent techniques for tracking the continuity of objects.

Obviously, failing to detect actin filaments for example due to limited length does not rule out their presence. In the future, technical improvements such as implementation of phase plate tomography [51] and better detectors, as well as improved algorithms for data mining and analysis could lead to a shift in detecting structures in noisy tomograms and circumvent the size limit apparently needed for the detection of short actin filaments. In this respect it is curious to note the presence of long filopodia-like projections from the apical end of some sporozoites, which intuitively suggest that they might be caused by the polymerization of actin filaments in the supra-alveolar space. Much larger extensions were present at the apical end of *T. gondii *tachyzoites when the F-actin stabilizing drug jasplakinolide was applied [27]. The bundles of long actin filaments were detected in such extensions. We failed to detect such structures in *Plasmodium *sporozoites when applying jasplakinolide (unpublished data) further suggesting that the way actin polymerization is controlled in these two parasites might differ in some important details. The material present within these extensions appeared similar with that in the supra-alveolar space. This would suggest that this structure is not strictly associated with the IMC, but is either soluble or associated to the plasma membrane, although such an association could be transient or unstable. However, no structural connectors could be found between the material and either the IMC or the plasma membrane, suggesting that the material is likely not tightly membrane bound. On the other hand the possible connecting molecules might be too rare or beyond the resolution of the method used in this study.

A third reason for not detecting filaments is the possibility that sporozoites imaged by cryogenic electron tomography were not motile at the time of freezing. If actin filaments only form at sites of adhesion to the substrate and are not formed anywhere else, this could lead to the absence of filaments in non-motile parasites, such as the ones that are not adhered to the surface of the EM grid. As we anticipated this problem, we aimed at establishing a correlative approach to first visualize motile sporozoites with the light microscope at ambient temperatures and then correlate these to images later recorded by cryo-light microscopy [52] and cryo-electron microscopy after plunge freezing the samples. This strategy allows correlation between light and electron microscopy for well adhering cells [37]. Unfortunately, sporozoites often appeared to be displaced from the substrate during blotting right before plunge-freezing and could rarely be located by cryo-light or the cryo-electron microscope at the same sites as before freezing [37]. However, even if we would have been successful in localizing motile sporozoites, filaments might only form at the contact sites of sporozoites to the grid, which unfortunately is the place yielding the lowest resolution in cryo-electron tomograms.

Lastly, it might be possible that during the blotting step just prior to plunge-freezing or during plunge-freezing itself the integrity of the plasma membrane might have been damaged. This could have led to a rapid depolymerization of the already short filaments. Indeed, we recently showed membrane leakage to occur in fibroblasts during blotting [37].

One possibility to circumvent these problems would be to image sporozoites lacking the sporozoite specific protein SPECT, which is needed for transmigration of cells [53,54]. These sporozoites efficiently invade cells and it might be possible to trap sporozoites during the process of invasion without displacing them during blotting as they are intimately associated with the host cell. However, the additional plasma membrane and cytoplasm of the host cell that surrounds the parasite during this step [54] might increase the thickness of the ice layer thus decreasing the quality of the tomograms, making it harder to reveal actin filaments. Indeed, we recently described the difficulty of obtaining full tomographic tilt series from the thicker central regions of isolated sporozoites [7]. To circumvent this problem, cryo-electron microscopy of vitrified sections (CEMOVIS) could be applied, which allows tomography of rapidly frozen and cryo-sectioned samples [55,56]. Alternatively, merozoites could be imaged in the process of invading an erythrocyte ghost [57,58]. However, even if actin filaments would be revealed, it would still be interesting to image actin filaments during sporozoite gliding as filaments might well be differently arranged during motility and host cell invasion.

Instead of using electron microscopy, it might also be feasible to identify actin filaments using optical nanoscopy methods [59,60], especially as these can now be performed with classic fluorescent proteins [61]. However, the maximum resolution of about 25 nm that these approaches routinely achieve might still be too little for identification of short actin filaments. It would be interesting for such an approach to first apply a similar simulation analysis as we present in Figures 7 and 8 prior to performing extensive imaging and image analysis. The use of proteins that bind only to F-actin but not to G-actin [62] could also be helpful in analyzing actin filaments during migration and invasion, possibly in combination with high resolution total internal reflection fluorescence microscopy [63].

In conclusion, by combining cryogenic electron tomography with *in silico *modeling we defined limits within which short filaments can be visualized in cells and discuss possible ways of circumventing these limits.

## 4. Materials and methods

### 4.1. Parasites and light microscopy

*Plasmodium berghei *(strain Nk65) sporozoites expressing cytoplasmic GFP were isolated from infected *Anopheles stephensi *salivary glands and imaged in serum free RPMI or phosphate buffered saline containing 3% bovine serum albumin [64]. Sporozoites were then transferred either on EM grids (see below) or onto glass slides. Imaging was performed on an inverted Axiovert 200 M Zeiss microscope in an air-conditioned imaging suite at room temperature (24°C). Images were collected with a Zeiss Axiocam HRm every 1 second using the Axiovision 4.6 software and 63× objective lens (NA 1.40). Images were processed using ImageJ and figures assembled using the Adobe Creative suite package.

### 4.2. Cryo-electron tomography

was performed essentially as described before [7,34]. *Plasmodium berghei *(strain Nk65) sporozoites were transferred onto EM carbon grids and incubated for 5-40 min. After removal of excess liquid by blotting with a filter paper, grids were rapidly plunged into liquid ethane and stored in liquid nitrogen. Grids were mounted in a Gatan cryo-holder (model: 626) and investigated using a cryo-electron microscope (FEI - CM 300 or FEI - Polara G2, both operating at the accelerating voltage of 300 keV, equipped with TWIN objective lens, field emission gun (FEG) and Gatan post column energy filter). The tilt series of low dose images (with a cumulative dose of under 10 000 electrons/nm^2^) were recorded on a 2048 pixel Gatan CCD camera, at a magnification of 43,000 (0,82 nm/pixel), and an objective lens defocus between -5 and -15 μm. We generally aimed at covering an angular range of -60° to 60° with 2° increment and filtered at zero energy loss. The recordings from the center of sporozoites frequently did not yield a full angle coverage [7]. For this study we used a subset from a total of 50 tomograms of tilt images aligned using fiducial gold markers. These reconstructions were calculated by weighted back-projection using the 'EM-image processing package' [65]. For visualization tomograms were filtered using non-linear anisotropic diffusion [66]. Visualization, volume rendering, and segmentation were performed using the Amira package (TGS Europe S.A., France). Quantitative analysis of tomograms was performed with the TOM toolbox for Matlab [67].

### 4.3. Simulations of image formation

Filaments were generated with 5 nm diameter and three different lengths. The lengths of filaments were normally distributed with a standard deviation of the half the length around the average of 24, 48 and 72 nm, respectively. Filaments were iteratively placed in random locations at random orientations between walls (IMC and the plasma membrane) spaced 30 nm away from each other (Figure 8). Filaments were placed such that they did not overlap with the walls and the other filaments. Iterative placement of filaments finished with reaching two final volumes: 5% ("high concentration", Figure 8, top) and 0.7% ("low concentration", Figure 8, bottom) of the total volume available between the membranes. For the simulation the volume of the voxel was 0.82^3 ^nm^3 ^as in most of the actual tomograms.

Special topologies of filaments were generated with an average length of 48 nm (Figure 7 top and middle row) and 30 nm (bottom row) and a "low concentration" of filaments. The simulation of the tomographic reconstruction procedure was performed similarly to those described previously [34] along the following steps:

1. The initial volume was tilted with 2° steps from -60° to +60° degrees; at every step the projection was acquired.

2. To every projection a randomly generated white noise was added in Fourier space. The amount of noise was σ_projection_/σ_noise_0.5 ("low noise") and 0.15 ("high noise"); σ is the standard deviation from the mean, which was set to 0 for both the noise and the projection.

3. Convolution with the contrast transfer function (CTF) in Fourier space. Parameters used for generation of the CTF function with "tom_ctf" from the TOM toolbox [67]: defocus: -10 μm, accelerating voltage: 300 keV, pixel size: 0.82^2 ^nm^2^; for all other parameters the default values were used.

4. Back projection of the projections to a three-dimensional volume (tomogram) customized in the TOM toolbox.

5. Volume rendered visualization using Amira 4. The threshold levels were calculated in a way that the number of pixels in the volume after reconstruction was the same as in the original volume. Views in figure 8, the top and middle columns in figure 7 are from the plane of the plasma membrane towards the IMC and perpendicular to the direction of the electron beam. Views in the third column in figure 7 are along the IMC and the plasma membrane in the direction of the electron beam.
